# Metabolic Markers Demonstrate the Heterogeneity of Myosteatosis in Community-Dwelling Older Black Men from the Health ABC Study

**DOI:** 10.3390/metabo11040224

**Published:** 2021-04-07

**Authors:** Samaneh Farsijani, Megan M. Marron, Iva Miljkovic, Mary Elizabeth Baugh, Stephen B. Kritchevsky, Anne B. Newman

**Affiliations:** 1 Department of Epidemiology, Graduate School of Public Health, University of Pittsburgh, Pittsburgh, PA 15261, USA; saf114@pitt.edu (S.F.); mmm133@pitt.edu (M.M.M.); miljkovici@edc.pitt.edu (I.M.); 2 Center for Aging and Population Health, University of Pittsburgh, Pittsburgh, PA 15213, USA; 3 Fralin Biomedical Research Institute, Virginia Polytechnic Institute and State University, Roanoke, VA 24016, USA; mebaugh@vtc.vt.edu; 4 Section on Gerontology and Geriatric Medicine, Department of Internal Medicine, Wake Forest School of Medicine, Winston-Salem, NC 27157, USA; skritche@wakehealth.edu

**Keywords:** myosteatosis, metabolomics, aging, heterogeneity, African American, mobility, muscle

## Abstract

Myosteatosis is a complex condition, associated with aging and diverse pathological conditions (e.g., diabetes), that contributes to mobility disability. Improved characterization of myosteatosis is required to develop targeted interventions to maintain muscle health in aging. We first determined the associations between plasma metabolites and intermuscular fat (IMF) in a cross-sectional analysis of 313 older Black men from Health ABC Study. Using partial correlation analysis, 34/350 metabolites were associated with IMF, the majority of which were lipids and organic acids. Next, we used Homeostasis Model Assessment of Insulin Resistance (HOMA-IR), as an indicator of metabolic health to delineate the anthropometric, functional, and metabolic heterogeneity of myosteatosis in a case-control matching analysis. We categorized participants based on their IMF and HOMA-IR levels into: Low-IMF with Low- versus High-HOMA, as well as High-IMF with Low- versus High-HOMA. Among participants with similar levels of IMF, those who were metabolically unhealthy, i.e., with High HOMA-IR, had higher fat and lean mass, muscle strength, and had hyperglycemia, hypertriglyceridemia, hyperinsulinemia, and higher levels of plasma metabolites belonging to diacylglycerols, triacylglycerols, fatty acid and aminoacyl-tRNA biosynthesis pathways versus those with Low HOMA-IR. In summary, HOMA-IR delineates the heterogeneity of myosteatosis by distinguishing metabolically healthy versus unhealthy individuals.

## 1. Introduction

Aging is associated with an increase in adipose tissue storage and redistribution of fat from subcutaneous to ectopic tissues, including muscles [1,2,3,4]. Increased intramyocellular (within myocytes) and intermuscular (within the fascia surrounding skeletal muscles) fat depots, known as myosteatosis, is associated with decreased muscle quality [5] and increased risk of mobility disability [6,7,8]. Muscle fat deposition is also seen in other pathological conditions, including cancer cachexia and spinal cord injury [9,10,11] as well as metabolic disorders (e.g., obesity and type-2 diabetes) [12,13]. However, the role of coexisting conditions in determining the health consequences related to myosteatosis has yet to be discovered in older adults. We have previously shown that myosteatosis is a heterogeneous phenotype by examining the relationship between midthigh intermuscular fat (IMF) area and physical function according to the muscle area in a cross-sectional analysis of Health, Aging, and Body Composition (Health ABC) study [14]. We showed that high IMF is associated with poor physical performance (i.e., low leg strength, slow walking speed, and poor chair stand performance) only in participants with high muscle area. Yet, the metabolic, anthropometric, and functional heterogeneity of myosteatosis needs to be further explored.

Insulin resistance has been used to delineate the heterogeneity of obesity through defining metabolically healthy versus unhealthy phenotypes among obese individuals [15,16]. Considering that an increase in muscle fat depots is associated with insulin resistance and increased risk of diabetes [17], we used the Homeostasis Model Assessment of Insulin Resistance (HOMA-IR) [18,19] as a validated metabolic marker to delineate the anthropometric, functional, and metabolic heterogeneity associated with myosteatosis. Characterizing subgroups of older adults with equal amounts of myosteatosis helps early identification of older adults at higher risk of developing functional impairments related to myosteatosis, including mobility disability.

We first determined the associations of plasma metabolites with IMF and HOMA-IR in a cross-sectional analysis of 313 community-dwelling older Black men from the Health ABC Metabolome Ancillary Pilot Study. Next, we performed a case-control analysis on individuals with similar levels of IMF, but different HOMA-IR levels to determine the anthropometric, functional, and metabolic heterogeneity of myosteatosis. We hypothesized that greater IMF and HOMA-IR levels would be associated with dysregulated lipid and protein metabolic profiles. We further hypothesized that older adults with the same levels of IMF, but different levels of HOMA-IR, would have distinct body composition, physical function, and metabolic profiles.

## 2. Results

### 2.1. Participant Characteristics

Table 1 shows participant characteristics according to quartiles of IMF. Participants in the higher quartiles were heavier and had higher total body lean mass, fat mass, midthigh muscle area and subcutaneous fat area (SFA). There were no differences in the percentage of energy intake from fat, carbohydrate, and protein or total calorie intake across IMF quartiles. Physical performance was comparable between IMF quartiles, except leg strength which was higher in participants in the higher IMF quartiles. Participants with higher IMF had higher levels of fasting blood glucose, insulin, HOMA-IR, triglycerides, total cholesterol, and LDL and marginally lower HDL levels (*p* = 0.080).

### 2.2. Metabolomics Profiling of Muscle Fat Deposition

**Figure 1:**
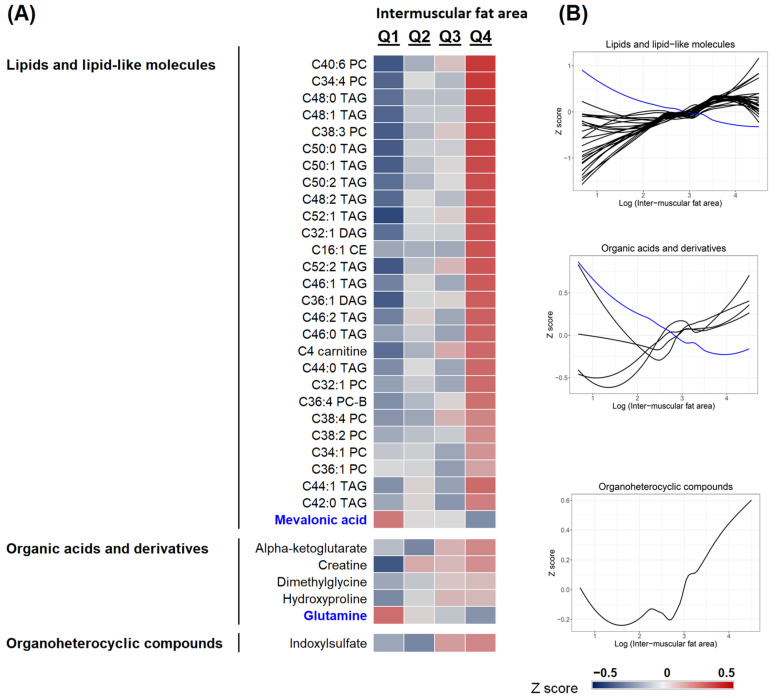
Superclass of 34 metabolites associated with intermuscular fat area. (**A**) Plasma levels of metabolites as standardized Z-scores across quartiles (Q) of intermuscular fat area. (**B**) Metabolites that were positively (in black) and negatively (in blue) correlated with intermuscular fat area after adjusting for age, weight, physical activity, total number of medications, and smoking with a false discovery rate of ≤0.25 to account for multiple comparisons.

Out of 350 metabolites, 161 metabolites were correlated with IMF (*p* < 0.05). After adjusting for age, weight, physical activity, total number of medications, and smoking, 34 metabolites remained significant with a false discovery rate of ≤0.25 to account for multiple comparisons. Figure 1A summarizes the plasma levels of these metabolites, as standardized Z-scores across quartiles of IMF. Metabolic profiles of participants were distinctly different across IMF quartiles with higher levels of lipids, organic acids, and organic heterocyclic compounds in participants in higher IMF quartiles compared to those with lower IMF. The majority of the metabolites associated with IMF were lipids and lipid-like molecules (28 out of 34), followed by organic acids, including amino acids (5 out of 34). Except glutamine (from organic acids) and mevalonic acid (from fatty acids) which were negatively correlated with IMF, the remaining metabolites were positively correlated with IMF (Figure 1B).

### 2.3. Metabolite Signature Associated with HOMA-IR

**Figure 2:**
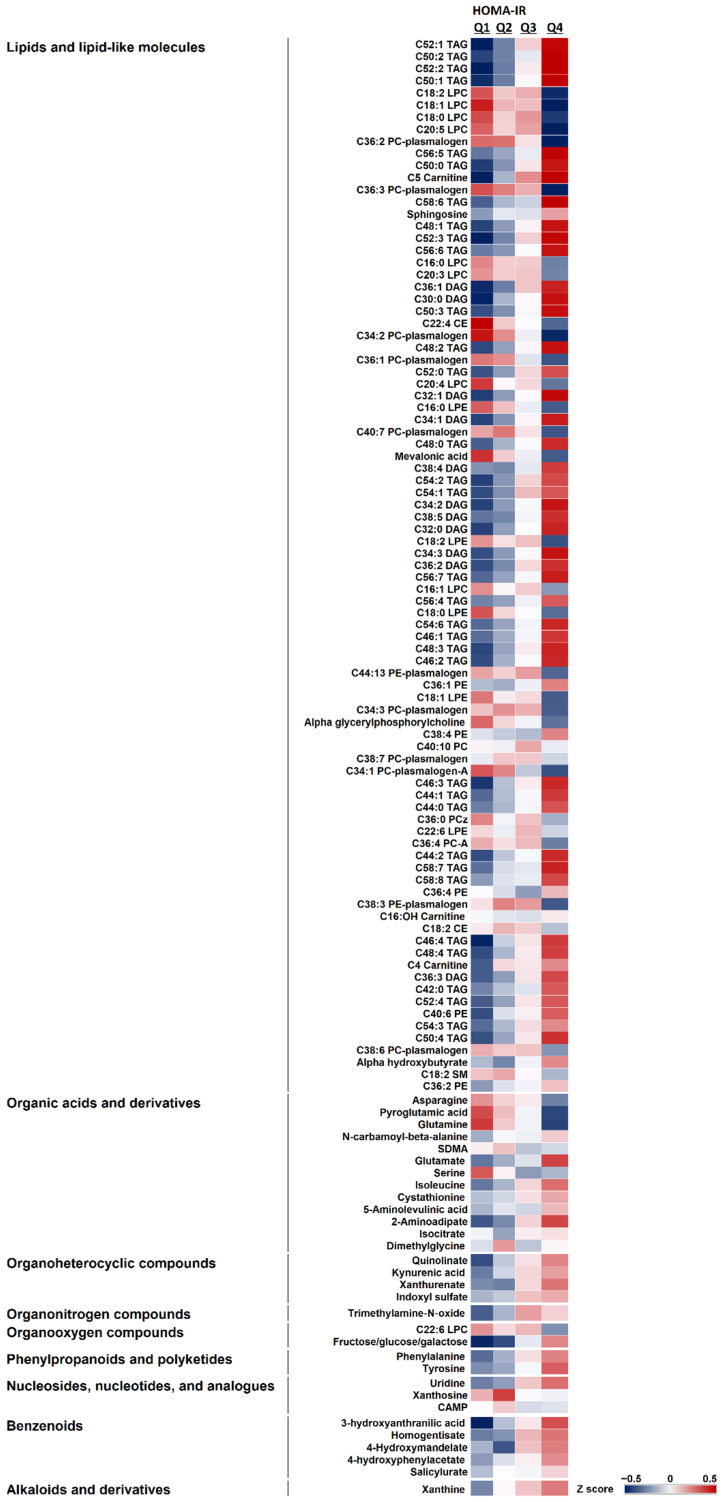
Representation of the 118 circulating metabolites associated with Homeostasis Model Assessment of Insulin Resistance (HOMA-IR) and their levels across HOMA-IR quartiles, after adjusting for age, weight, physical activity, medications, and smoking and accounting for multiple comparisons (false discovery rate 0.25).

We identified 118 circulating metabolites that were associated with HOMA-IR, after adjusting for age, weight, physical activity, number of medications, and smoking and accounting for multiple comparisons (false discovery rate ≤ 0.25). Figure 2 compares the plasma levels of these metabolites across HOMA-IR quartiles. The majority of metabolites belonged to lipids and lipid-like molecules (74%), followed by organic acids (11%), and remaining metabolites (15%) were from other metabolic subclasses, including benzenoids, organic heterocyclic, and nucleotides. Most metabolites (66%) were positively correlated with HOMA-IR (all of the metabolites in organic heterocyclic, organonitrogen, phenylpropanoids and polyketides, alkaloids, and benzenoids superclass and majority of lipids), while the rest (34%) were negatively correlated.

### 2.4. The Heterogeneity of IMF

To delineate the heterogeneity associated with IMF, we compared body composition, physical function, plasma biomarkers and metabolites among participants with similar levels of IMF but different levels of HOMA-IR. Participant characteristics across our matched case-control groups are shown in Table 2. Participants within low and high IMF groups were well matched for IMF levels (*p* > 0.05) but had significantly different HOMA-IR levels (*p* < 0.05; Table 2).

#### 2.4.1. Body Composition

Participants in High HOMA-IR subgroups, regardless of their IMF levels, were heavier and had higher total body lean, fat mass and midthigh muscle area compared to those in Low HOMA-IR subgroups (Table 2). Additionally, within High IMF groups, those in High HOMA-IR subgroup had higher lean mass and appendicular lean mass (ALM) compared to Low HOMA-IR subgroup, while within Low IMF groups, lean mass was comparable, regardless of the HOMA-IR levels.

#### 2.4.2. Physical Activity and Performance

Physical activity levels were not different between IMF groups, regardless of HOMA-IR levels. Within High IMF groups, participants in High HOMA-IR subgroup had higher grip and leg (i.e., isokinetic torque) strength compared to those in Low HOMA-IR subgroup, while muscle strength was similar within Low IMF groups (Table 2). Torque normalized to mid-thigh muscle area (i.e., specific torque) was comparable across our matched case-control groups (Table 2). Within Low IMF group, participants in the High HOMA-IR subgroup walked faster compared to their matched controls, while within High IMF group walking speed was not different in High HOMA-IR versus Low HOMA-IR subgroups (Table 2).

#### 2.4.3. Blood Biomarkers

Participants in High HOMA-IR subgroups, regardless of their IMF levels, had higher fasting plasma insulin, glucose, triglycerides, and lower HDL levels, compared to those in the Low HOMA-IR subgroups (*p* < 0.01; Table 2).

#### 2.4.4. Plasma Metabolite Profiles

**Figure 3:**
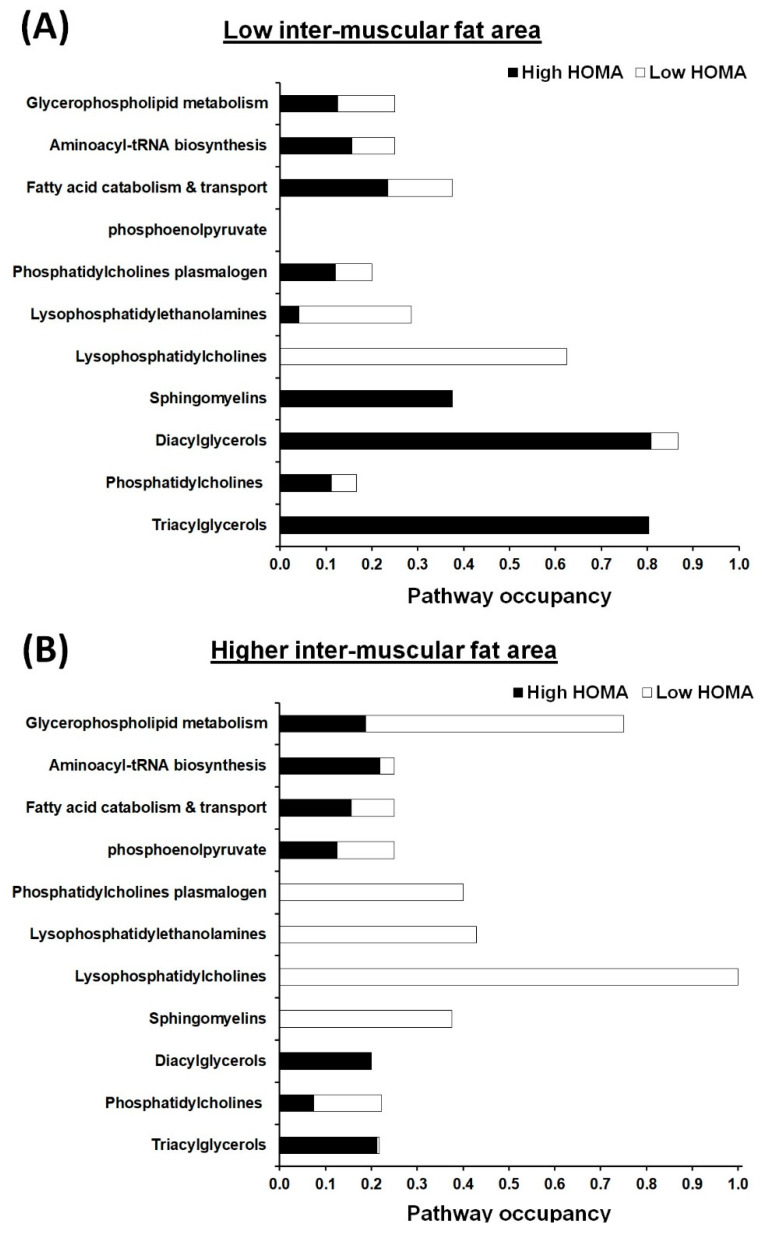
Pathway occupancy rates of statistically different metabolites between high and low HOMA-IR groups, in participants with the same levels of intermuscular fat ((**A**) Low intermuscular fat area and (**B**) high intermuscular fat area). Black bars, the ratio of metabolites higher in High HOMA-IR than in low HOMA-IR; white bars, the ratio of metabolites higher in low HOMA-IR than in High HOMA-IR.

Figure 3 shows pathway occupancy rates of significantly different metabolites between High and Low HOMA-IR subgroups in participants with the same levels of IMF. We first categorized 172 metabolites that were significantly different (false discovery rate ≤ 0.25) across our matched case-control groups based on their metabolic pathway or biofunction (if unknown) and then selected metabolic pathways represented by more than four metabolites to construct pathway occupancy (data not shown). Overall, 131 metabolites belonging to 11 metabolic pathways were compared within IMF groups (Figure 3). Regardless of IMF levels, the majority of metabolites in diacylglycerol, triacylglycerol, fatty acid catabolism and transport, and aminoacyl-tRNA biosynthesis pathways belonged to the High HOMA-IR subgroup, while majority of metabolites in lysophosphatidylcholines pathway belonged to the Low HOMA-IR subgroups.

Within Low IMF groups, diacylglycerols and triacylglycerols scored ≥80% of pathway occupancy and majority of metabolites belonged to the High HOMA-IR compared to the Low HOMA-IR subgroup (Figure 3A). Additionally, lysophosphatidylcholine metabolism scored more than 65% of pathway occupancy, with almost all the metabolites higher in the Low HOMA-IR subgroup. Similarly, lysophosphatidylethanolamines scored 30% of pathway occupancy with the majority of metabolites being higher in the Low HOMA-IR subgroup compared to the High HOMA-IR (Figure 3A).

Within High IMF groups, lysophosphatidylcholines scored 100% of pathway occupancy, with all metabolites belonging to the Low HOMA-IR compared to the High HOMA-IR subgroup (Figure 3B). Additionally, glycerophospholipid metabolism scored more than 70% of pathway occupancy, with the majority being Low HOMA-IR-enriched metabolites. Phosphatidylcholines plasmalogen, lysophosphatidylethanolamines, and sphingomyelins metabolic pathways scored more than 35% occupancy and consisted of mainly Low HOMA-IR-enriched metabolites.

## 3. Discussion

Our results showed that higher IMF was associated with higher body weight, total lean, fat mass, and midthigh muscle area as well as higher muscle strength in community-dwelling older Black men. Higher IMF levels were also associated with dysregulated glucose and lipid metabolism and insulin resistance. After controlling for potential confounders, 34 plasma metabolites (out of 350) were associated with IMF, the majority of which were lipids and organic acids and positively correlated with IMF. Interestingly, among participants with similar levels of IMF, those who were metabolically unhealthy, defined by high HOMA-IR levels, had higher total lean and fat mass, muscle area, strength, and had hyperglycemia, hypertriglyceridemia, hyperinsulinemia, and disrupted lipid metabolism compared to those with low HOMA-IR levels.

### 3.1. Age-Related Increase in Skeletal Muscle Fat Infiltration Is Associated with Dysregulated Lipid Metabolism

Myosteatosis in aging muscles is related to multiple health conditions, including mobility disability, diabetes, and increased risk of mortality [20]. However, pathophysiology and metabolic changes associated with myosteatosis remains largely unknown. To our knowledge, no study has addressed the differences in plasma metabolites in relation to myosteatosis in older adults. Here, we showed that higher IMF areas were associated with higher levels of plasma lipids and organic acids (e.g., alpha-ketoglutarate and creatine) suggestive of dysregulated metabolism of lipids and lipid-like molecules as well as organic acids. The observed elevation in lipid and organic acid metabolites in plasma was in line with higher plasma levels of triglycerides, total cholesterol, LDL, and lower HDL levels among participants with higher IMF values. These findings are consistent with reported metabolic perturbations associated with myosteatosis, including reduced lipolysis and lipid oxidation [2,21] leading to fat accumulation within muscles, such as triglyceride deposition, which is associated with increased risk of metabolic diseases (e.g., diabetes) [22].

### 3.2. Body Composition, Physical Performance, and Metabolic Heterogeneity of Myosteatosis

We have previously shown the heterogeneity associated with myosteatosis in a cross-sectional analysis of the Health ABC study by demonstrating that the negative association between IMF and physical performance is only observed in individuals with high midthigh muscle area [14]. However, the metabolic and phenotypic heterogeneity associated with muscle fat depots needs to be further determined.

Various metabolic measures, including HDL, triglycerides, and HOMA-IR have been suggested and utilized to delineate heterogeneity of obesity through defining metabolically healthy versus unhealthy phenotypes among obese individuals [23,24,25]. Hamer et al. (2012) [24] in a longitudinal analysis of 22,203 men and women (aged 54.1 ± 12.7 year) showed that regardless of BMI levels, abnormal blood biomarkers were predictive of mortality risk. They showed that metabolically unhealthy obese participants (defined by high blood pressure, HDL, diabetes, waist circumference, and C-reactive protein) were at higher risk of all-cause mortality compared with their metabolically healthy obese counterparts. Considering that increased muscle fat depots is associated with insulin resistance [1,17], we assessed if HOMA-IR, as a validated indicator of insulin resistance, can delineate the heterogeneity associated with myosteatosis. In this study, we were able to identify metabolically healthy individuals (with low HOMA-IR and low plasma lipids, glucose, and insulin levels) versus metabolically unhealthy irrespective of their IMF levels. Moreover, participants with high HOMA-IR had distinct body composition characteristics, including higher leanness, fat mass, and thigh muscle area compared to those with low HOMA-IR, regardless of their IMF levels.

Using HOMA-IR, we also showed the heterogeneity in physical performance among participants with similar levels of IMF. In participants who had equally high IMF levels, those with high HOMA-IR had higher grip and leg strength compared to those with low HOMA-IR levels, which was related to their higher muscle size since specific torque was comparable across our matched case-control subgroups. Additionally, among participants who had equally low IMF levels, high HOMA-IR was associated with faster walking speed. Additionally, metabolomic profiling of our matched case-control groups showed that, regardless of IMF levels, all the metabolites related to lysophosphatidylcholine and diacylglycerols pathways were higher in Low HOMA-IR and High HOMA-IR subgroups, respectively. Notably, lipogenic effects of insulin, unlike its effects on glucose uptake, have been reported to be maintained in individuals who have insulin resistance [26]. Therefore, dysregulated lipid metabolism and lipid metabolites observed among participants with high HOMA-IR could be secondary to insulin resistance. Identifying metabolites or pathways that cause or mitigate myosteatosis may provide further insight into metabolic mechanisms that lead to muscle dysfunction in aging.

### 3.3. Clinical Implications of Heterogeneity of Myosteatosis

Identifying novel methods of phenotyping myosteatosis will allow for characterization of metabolically healthy versus unhealthy myosteatosis and prediction of its adverse health outcomes. Of note, older adults with so-called metabolically healthy myosteatosis may still be at higher risk of negative health outcomes associated with myosteatosis compared to individuals who do not experience muscle fat infiltration. Moreover, characterization of metabolically healthy versus unhealthy myosteatosis may ultimately promote individualized and targeted lifestyle interventions to improve aging outcomes. For example, individuals with metabolically unhealthy myosteatosis are characterized by higher body fat storage as well as higher plasma levels of metabolites involved in lipid metabolism in addition to hyperlipidemia, hypertriglyceridemia, and hyperinsulinemia. Therefore, these individuals may benefit more from calorie restriction/weight reduction and exercise. Calorie restriction and weight reduction in older adults with metabolically healthy myosteatosis who have less muscles may exacerbate muscle wasting. Hence, these individuals may benefit more from protein supplementation and strength training to maintain their muscle tissues.

### 3.4. Strength and Limitations

This study is the first to determine metabolomics profiling of muscle fat deposition as well as disclosing the heterogeneity in body composition and metabolism associated with myosteatosis in older individuals, using a subset of older adults from the well-characterized Health ABC study with available rich metabolomics data. In this study we used data from the Health ABC Metabolome Ancillary Study which was a small pilot study performed only on Black men [27]. Therefore, generalizability of our findings to women and other racial groups is limited.

In summary, IMF was associated with dysregulated lipid metabolism, but insufficiently demonstrated the variations in body composition, mobility function, physical performance, and metabolic health between older individuals. Using HOMA-IR as a metabolic indicator allowed us to identify individuals who were metabolically healthy from those who were unhealthy, regardless of IMF levels. Notably, individuals with similar levels of IMF but different levels of HOMA-IR had distinct anthropometric and functional status, highlighting the role of metabolic profiling in determining the health consequences associated with myosteatosis. Characterizing subgroups of individuals with equal amounts of muscle fat depots can promote early identification of older individuals who are at higher risk of developing adverse health outcomes related to myosteatosis, including disability.

## 4. Materials and Methods

### 4.1. Study Population

In the original Health ABC cohort, 3075 participants (51.5% women and 41.7% Black), aged 70–79 years at baseline (1997–1998) were enrolled from Memphis, TN, and Pittsburgh, PA [28]. Eligible participants had no difficulty walking a quarter of a mile, climbing 10 steps, and performing basic activities of daily living, and did not use ambulatory assistive devices for walking. Participants were ineligible if they had active cancer during the past three years or planned on moving from the study sites within the next three years. All participants provided written informed consent and the study was approved by Institutional Review Board of the Universities of Pittsburgh and Tennessee (ethical approval code: IRB960212).

**Figure 4:**
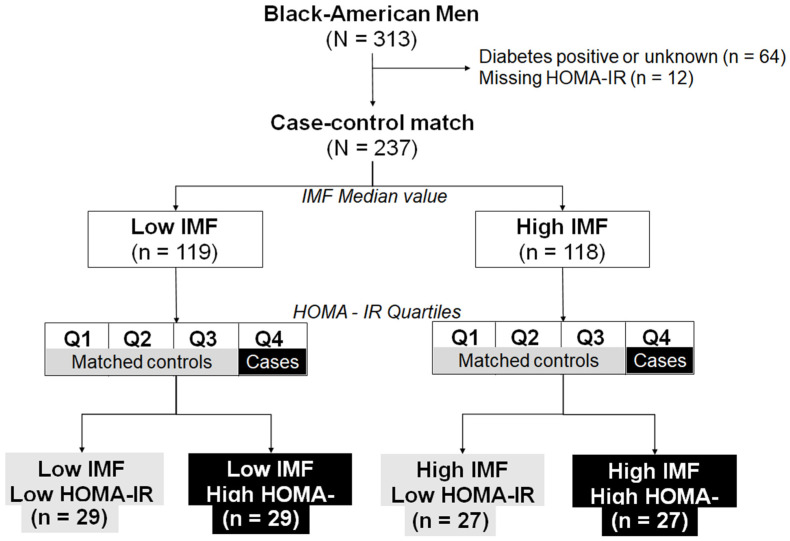
Case-control matching analysis to identify participants with similar levels of IMF but different levels of HOMA-IR. Considering that study participants had the same sex, race, and similar age, matching was performed based on only IMF areas (±1 cm^2^; 1:1 allocation ratio). HOMA-IR, Homeostasis Model Assessment of Insulin Resistance; IMF, intermuscular fat area; Q, quartile.

In this study, we used data from the Health ABC Metabolome Ancillary Pilot Study on 313 older Black men who were randomly selected from the year two visit [27] (approved analysis plan reference # AP20–1521). The Health ABC Metabolome Ancillary Pilot Study was conducted to identify biomarkers of lean and fat mass in older Black men who were randomly selected (using stratified random sampling by sex, race, and available blood samples without replacement) from the original Health ABC study [27]. The ancillary study was limited to Black Americans (due to the higher prevalence of obesity-related diseases and their higher muscle mass compared to Caucasian Americans) and male participants to limit sex-differences in body composition [27]. First, we conducted a cross-sectional analysis to determine the association of plasma metabolites with IMF and HOMA-IR among all participants. In addition to Health ABC inclusion criteria, in our study we included participants with available IMF measurements. Next, we conducted a case-control matching analysis (Figure 4) to delineate the anthropometric, functional, and metabolic heterogeneity associated with muscle fat deposition. We initially excluded participants with diabetes (n = 63) and missing HOMA-IR data (n = 13). Then, we used the median value of IMF to categorize participants into low and high IMF groups. Next, within each IMF category, we determined quartiles of HOMA-IR and selected participants in the highest quartile as our cases and identified matched controls from the other quartiles, using IMF areas (±1 cm^2^; 1:1 allocation ratio) as the matching factor. Therefore, our study groups consisted of participants with the similar levels of IMF but different levels of HOMA-IR (i.e., low IMF and low HOMA-IR (n = 29) or high HOMA-IR (n = 29); in addition to high IMF and low HOMA-IR (n = 27) or high HOMA-IR (n = 27) (Figure 4)).

### 4.2. Measurement of Plasma Metabolites

We measure 350 plasma metabolites in overnight fasting blood samples collected at year two, as previously described [29]. In brief, using a non-targeted metabolomic approach, plasma samples stored at −80 °C since the time of collection (1998–1999) were processed by LC-MS to measure polar metabolites (e.g., amino acids, dipeptides, sugars, and purines) and lipids (e.g., triglycerides). Metabolite values are LC-MS peak areas, analyzed using TraceFinder (ThermoFisher Scientific, Waltham, MA, USA) and Progenesis QI (Nonlinear Dynamics, Borehamwood, UK).

### 4.3. Body Composition Assessment

Dual-energy X-ray absorptiometry (DXA) (QDR 4500A; Hologic Inc., Waltham, MA, USA) was used to determine whole body lean mass and fat mass at baseline, as previously reported [30]. Cross-calibration phantoms were used to assess the reliability of DXA measures and adherence to the study protocol at clinical sites. Appendicular lean mass (aLM) was determined as sum of the non-bone lean mass in arms and legs. Height (by Harpenden stadiometer, Holtain Ltd., Crosswell, UK) and body weight (by calibrated balance-beam scale) were measured wearing a hospital gown without shoes and used to calculate body mass index (BMI; kg/m^2^).

### 4.4. Midthigh Cross-Sectional Area Assessment

Midthigh muscle cross-sectional area (cm^2^), subcutaneous fat area (SFA, cm^2^), and intermuscular fat area (IMF, cm^2^) were determined at baseline by computed tomography (CT), as described before [14]. A single 10 mm axial image was taken of the mid-thigh cross-sectional area (the midpoint of the distance between the medial edge of the greater trochanter and the intercondyloid fossa). Next, a software (RSI Systems, Boulder, CO, USA) was used to quantify IMF as the area of fat density within the deep fascial plane surrounding the thigh muscles as well as the muscle area as the total area of nonfat and nonbone tissues within the fascial border [14].

### 4.5. Dietary Assessment

Usual nutrient intake was determined by a 108 item interviewer-administered food frequency questionnaire (FFQ), developed for the Health ABC study (Block Dietary Data Systems, Berkeley, CA, USA) [31]. Standard kitchen measures, food models, wood blocks, and flash cards were used to aid participants in estimating portion sizes. Collected FFQs were analyzed by Block Dietary Data Systems to estimate macro- and micro-nutrient content of the reported foods.

### 4.6. Physical Performance

Maximum grip strength at baseline was determined by hand-held dynamometer (Jaymar, Bolingbrook, IL, USA) out of the two trial on the right hand. Maximum isokinetic muscle torque of the knee extensors was assessed by Kin-Com dynamometer (model 125 AP; Chattanooga, TN, USA), as described previously [32]. A knee extension test was performed on the right leg, unless contraindicated. Participants with history of stroke, uncontrolled hypertension, bilateral knee replacement, or severe bilateral knee pain were excluded from the test [33,34]. Peak torque was used to calculate muscle-specific torque per mid-tight muscle cross-sectional area (N.m/cm^2^) as an index of muscle quality [35]. Lower extremity function was assessed by fast six meter gait speed (i.e., walking speed (m/s) over 6 m), 20-meter gait speed (i.e., walking speed (m/s) over 20 m) and five repeated timed chair stands (number/sec, assigning 0 to participants who were unable to complete the test), and standing balance (0–90 sec) [32].

### 4.7. Blood Biochemistry

Plasma levels of total, high-density lipoprotein (HDL), and low-density lipoprotein (LDL) cholesterol (mg/dL), triglycerides (mg/dL) (Vitros 950 analyzer; Johnson & Johnson, New Brunswick, NJ, USA), glucose (mg/dL; YSI 2300 Glucose Analyzer; Yellow Springs, OH, USA), and insulin (μU/mL; Pharmacia, Uppsala, Sweden) were determined in fasting (≥8 h) blood samples drawn during baseline clinic visit. Insulin resistance was defined by HOMA-IR, using the following formula: [fasting glucose (nmol/L) × fasting insulin level (µU/mL)/22.5] [18,19].

### 4.8. Other Covariates

Participants self-reported their age, smoking habits, and brought all prescription medications to baseline clinic visit to determine total number of medications. Weekly physical activity levels were estimated by self-reported time spent on walking and stairs and converted to kilocalories/kilogram/week [36].

### 4.9. Statistical Analysis

Baseline characteristics of study participants were compared between quartiles of IMF, using independent samples Kruskal–Wallis test and one-way analysis of variance (ANOVA). Plasma metabolites were log-transformed and standardized. Partial correlation analysis was performed to determine plasma metabolites that are associated with IMF and HOMA-IR, while controlling for baseline age, weight, physical activity level, smoking, and medications. Multiple comparisons were accounted for using a Benjamini–Hochberg method [37] with a liberal false discovery rate of 0.25, as our analysis were exploratory.

Next, to disclose heterogeneity associated with IMF, we compared body composition, physical function, and blood biomarkers across our case-control matched groups, using ANOVA or independent samples Kruskal–Wallis test (for between group comparisons) and independent sample *t*-test or Mann–Whitney U test (for within group comparisons). To compare plasma metabolites within cases and controls, we used pathway occupancy analysis [38,39]. That is, we grouped metabolites that were significantly different across our matched case-control groups based on their metabolic pathways or function (in unknown) and selected pathways represented by more than four metabolites. Next, we compared metabolites within IMF groups, using independent *t*-test and when the *p* value was <0.05, we assigned a score of 1 and when *p* > 0.05, we assigned a score of 0 to that metabolite. Finally, obtained scores were divided by the number of metabolites within a specific pathway to determine the ratio of occupied metabolites that reached statistical significance level within a pathway. For each pathway, we also calculated percent contribution of metabolites to each matched case-control groups. All analyses were conducted using RStudio (version 1.3.959, RStudio, PBC, Boston, MA, USA) and SPSS Statistics (version 26.0 for Windows, IBM, Chicago, IL, USA).

## Figures and Tables

**Table 1:** Participant characteristics according to quartiles of intermuscular fat area.

IMF Quartiles	Q1 ≤11.81 cm^2^	Q2 11.82–18.01 cm^2^	Q3 18.02–27.11 cm^2^	Q4 ≥27.12 cm^2^	*p*
	(n = 78)	(n = 78)	(n = 79)	(n = 78)	
Age, y	73.9 ± 2.8	73.1 ± 2.6	73.5 ± 2.9	73.4 ± 2.8	0.341
Medications, n	4.5 ± 3.4	4.6 ± 4.8	4.8 ± 3.1	5.2 ± 4.0	0.212
**Body composition**					
Weight, kg	68.0 ± 9.4	77.2 ± 9.8	83.6 ± 9.6	95.8 ± 13.0	<0.001 ^§^
BMI, kg/m^2^	23.4 ± 3.1	25.7 ± 3.1	28.0 ± 2.9	31.4 ± 3.8	<0.001 ^§^
Total fat mass, kg	16.1 ± 4.7	20.8 ± 5.0	24.7 ± 4.3	31.2 ± 7.0	<0.001
Total lean mass, kg	49.2 ± 6.2	53.6 ± 6.0	56.1 ± 6.4	61.5 ± 7.2	<0.001 ^§^
ALM, kg	22.2 ± 3.3	24.4 ± 3.2	25.3 ± 3.2	28.3 ± 3.9	<0.001 ^§^
Muscle area, cm^2^	251.3 ± 44.7	274.5 ± 41.1	280.9 ± 41.7	311.0 ± 50.1	<0.001 ^§^
IMF, cm^2^	8.5 ± 2.3	14.9 ± 1.6	22.0 ± 2.7	39.4 ± 13.6	<0.001
SFA, cm^2^	71.1 ± 30.3	84.5 ± 31.1	104.6 ± 33.3	126.8 ± 39.9	<0.001 ^§^
**Diet**					
Energy, kcal/d	2272 ± 1102	2171 ± 920	2229 ± 1030	2038 ± 784	0.740
Fat intake, %kcal/d	34.8 ± 8.0	35.2 ± 6.7	34.4 ± 7.2	34.1 ± 8.0	0.810 ^§^
Protein intake, %kcal/d	13.2 ± 2.7	13.8 ± 3.0	14.6 ± 3.4	13.9 ± 3.2	0.075 ^§^
CHO intake, %kcal/d	53.3 ± 10.2	51.9 ± 8.2	52.0 ± 8.7	52.5 ± 9.1	0.771 ^§^
**Physical activity & performance**					
PA, kcal/kg/wk	78.5 ± 62.3	95.9 ± 93.5	76.5 ± 69.0	84.2 ± 79.4	0.703
Fast 6-m walk, m/s	1.16 ± 0.21	1.15 ± 0.22	1.15 ± 0.20	1.14 ± 0.17	0.979 ^§^
Chair stand, sec	0.36 ± 0.12	0.35 ± 0.11	0.35 ± 0.11	0.34 ± 0.11	0.547
Balance, 0–90	71.9 ± 23.4	69.5 ± 23.3	72.7 ± 19.9	73.9 ± 20.0	0.708
Grip, N	385.1 ± 105.7	409.9 ± 102.3	410.1 ± 99.8	422.5 ± 116.4	0.231 ^§^
Leg strength, N.m	126.9 ± 35.2	140.0 ± 38.1	139.0 ± 34.3	146.1 ± 38.5	0.022 ^§^
**Blood biochemistry**					
Glucose, mg/dL	96.5 ± 18.4	106.5 ± 32.4	112.8 ± 44.2	113.1 ± 36.0	0.002
Insulin, μIU/mL	6.18 ± 6.46	7.63 ± 4.58	7.92 ± 3.94	10.33 ± 5.04	<0.001
HOMA-IR	1.48 ± 1.75	1.99 ± 1.38	2.18 ± 1.46	2.70 ± 1.51	<0.001
Triglycerides, mg/dL	102.4 ± 60.0	118.2 ± 51.8	118.3 ± 64.4	121.9 ± 51.3	0.003
Total cholesterol, mg/dL	185.3 ± 31.6	202.9 ± 36.0	190.5 ± 34.2	193.3 ± 36.8	0.022
HDL, mg/dL	55.1 ± 14.7	51.3 ± 15.0	51.7 ± 13.9	49.9 ± 15.2	0.080
LDL, mg/dL	110.3 ± 32.0	128.4 ± 34.4	115.3 ± 31.7	119.0 ± 33.9	0.017

Notes: Mean ± standard deviation. *p* values by Independent samples Kruskal–Wallis test unless otherwise indicated. ^§^ One-way ANOVA. Q, Quartiles; ALM, appendicular lean mass; IMF, intermuscular fat area; SFA, subcutaneous fat area; CHO, carbohydrate; PA, physical activity; sec, second; N, Newton; N.m, Newton-meter; HOMA-IR, Homeostatic Model Assessment for Insulin Resistance; HDL, high-density lipoproteins; LDL, low-density lipoproteins.

**Table 2:** Body composition, physical function, and blood biochemistry of older adults with the same level of intermuscular fat area but different levels of insulin sensitivity (resistance versus sensitive).

	Low IMF Low HOMA	Low IMF High HOMA	*P*_Within_	High IMF Low HOMA	High IMF High HOMA	*P*_Within_	*P*_Between_
	(n = 29)	(n = 29)		(n = 27)	(n = 27)		
Age, y	73.3 ± 2.7	72.8 ± 2.6	0.436 ^¥^	73.9 ± 2.8	73.1 ± 2.4	0.333 ^Ɫ^	0.475
**Body composition**							
Weight, kg	70.6 ± 9	76.2 ± 8.2	0.017 ^¥^	85.6 ± 15.2	96.5 ± 13.5	0.008 ^¥^	<0.001 ^§^
Height, m	1.74 ± 0.06	1.71 ± 0.06	0.099 ^¥^	1.72 ± 0.07	1.75 ± 0.08	0.101 ^¥^	0.092 ^§^
BMI, kg/m^2^	23.4 ± 2.4	26.2 ± 2.7	<0.001 ^¥^	29.0 ± 4.5	31.4 ± 3.3	0.016 ^Ɫ^	<0.001 ^§^
Total fat mass, kg	16.8 ± 3.3	21.4 ± 4.0	<0.001 ^Ɫ^	26.2 ± 7.3	31.5 ± 7.0	0.010 ^Ɫ^	<0.001
Total lean mass, kg	51.2 ± 6.8	52.2 ± 5.5	0.536 ^¥^	56.9 ± 8.3	62.0 ± 7.7	0.025 ^¥^	<0.001 ^§^
ALM, kg	23.5 ± 3.8	23.3 ± 2.8	0.743 ^¥^	26.1 ± 4.4	28.9 ± 4.4	0.022 ^¥^	<0.001 ^§^
Muscle area, cm^2^	253.3 ± 35.8	271.8 ± 38.9	0.066 ^¥^	286.8 ± 53.5	326.8 ± 49.9	0.006 ^¥^	<0.001 ^§^
IMF, cm^2^	12.8 ± 3.0	12.8 ± 3.0	0.966 ^¥^	33.7 ± 15.5	36.0 ± 15.0	0.337 ^Ɫ^	<0.001
SFA, cm^2^	73.8 ± 24.6	90.6 ± 30.4	0.025 ^¥^	113.4 ± 44.3	127.5 ± 30.3	0.178 ^¥^	<0.001 ^§^
**Physical activity & performance**							
PA, kcal/kg/wk	113.9 ± 90.4	77.5 ± 88.4	0.053 ^Ɫ^	104.6 ± 92.3	79.8 ± 97.3	0.135	0.096
Fast 6-m walk, m/s	1.09 ± 0.24	1.20 ± 0.18	0.049 ^¥^	1.12 ± 0.17	1.15 ± 0.19	0.474 ^¥^	0.161 ^§^
20-m walk, m/s	1.24 ± 0.27	1.35 ± 0.18	0.090 ^¥^	1.30 ± 0.19	1.36 ± 0.25	0.222 ^Ɫ^	0.207
Chair stand, sec	0.34 ± 0.10	0.37 ± 0.09	0.269 ^¥^	0.35 ± 0.10	0.34 ± 0.11	0.959 ^Ɫ^	0.600
Balance, 0–90	67.3 ± 20.5	71.2 ± 24.2	0.370 ^Ɫ^	75.3 ± 15.8	73.9 ± 16.6	0.758 ^Ɫ^	0.615
Grip, N	387.0 ± 90.9	430.0 ± 116.6	0.163 ^¥^	377.4 ± 82.7	467.6 ± 120.9	0.006 ^¥^	0.016 ^§^
Torque, N.m	131.7 ± 31.6	142.7 ± 42.4	0.309 ^¥^	130.1 ± 31.3	159.7 ± 41.0	0.010 ^¥^	0.034
Specific torque, N.m/cm^2^	1.02 ± 0.20	1.02 ± 0.24	0.901 ^Ɫ^	0.91 ± 0.24	0.97 ± 0.21	0.326 ^¥^	0.239
**Blood Biochemistry**							
Glucose, mg/dL	90.8 ± 15.4	108.1 ± 20.6	0.001 ^Ɫ^	88.3 ± 9.8	107.2 ± 16.4	<0.001 ^Ɫ^	<0.001
Insulin, μIU/mL	3.33 ± 1.41	12.78 ± 8.13	<0.001 ^Ɫ^	5.72 ± 2.63	15.91 ± 2.90	<0.001 ^Ɫ^	<0.001
HOMA-IR	0.74 ± 0.32	3.38 ± 2.23	<0.001 ^Ɫ^	1.27 ± 0.65	4.23 ± 1.17	<0.001 ^Ɫ^	<0.001 ^§^
Triglycerides, mg/dL	95.8 ± 31.8	150.3 ± 82.3	0.002 ^¥^	98.0 ± 31.9	138.4 ± 49.2	0.001 ^¥^	<0.001 ^§^
TChol, mg/dL	194.4 ± 36.5	201.5 ± 29.2	0.414 ^¥^	193.2 ± 37.3	180.9 ± 29.6	0.183 ^¥^	0.227
HDL, mg/dL	57.6 ± 12.9	47.1 ± 13.2	0.003 ^¥^	55.8 ± 15.2	45.9 ± 10.7	0.005 ^Ɫ^	<0.001
LDL, mg/dL	117.7 ± 32.7	126.1 ± 28.7	0.305 ^¥^	117.9 ± 36.8	107.3 ± 24.1	0.217 ^¥^	0.158

Mean ± standard deviation. P_Between_ by independent samples Kruskal–Wallis test unless otherwise indicated. ^§^ One-way ANOVA. P_within_ by independent sample *t*-test (^¥^) and independent samples Mann–Whitney U test (^Ɫ^). ALM, appendicular lean mass; IMF, intermuscular fat area; SFA, subcutaneous fat area; CHO, carbohydrate; PA, physical activity; sec, second; N, Newton; N.m, Newton-meter; HOMA-IR, Homeostatic Model Assessment for Insulin Resistance; TChol, total cholesterol; HDL, high-density lipoproteins; LDL, low-density lipoproteins.

## Data Availability

The data presented in this study are available in article.
